# Concurrent CML and MBL in a COPD patient: a case of collision hematologic malignancies and opportunistic infection

**DOI:** 10.3389/fmed.2026.1872985

**Published:** 2026-08-21

**Authors:** Liang Zhang, Mei ying Zhang, Chang sheng Zhou, Xing yuan Ci, Long ying Zhu, Qi lei Hu, Zhi rong Qi, Shui Fu, Zuo jie Li

**Affiliations:** 1 Department of Clinical Laboratory, First People’s Hospital of Linping District, Hangzhou, Zhejiang, China; 2 Department of Pathology, Adicon Medical Laboratory Co., Ltd., Hangzhou, Zhejiang, China; 3 Departments of Respiratory and Critical Care Medicine, The People’s Hospital of Cangnan Zhejiang, Wenzhou, Zhejiang, China; 4 Department of Integrative Oncology, China-Japan Friendship Hospital, Beijing, China; 5 Department of Clinical Laboratory, The People’s Hospital of Cangnan Zhejiang, Wenzhou, Zhejiang, China

**Keywords:** chronic myeloid leukemia, clinico-laboratory dissociation, collision hematologic malignancies, COPD, monoclonal b-cell lymphocytosis, *Pneumocystis jirovecii* pneumonia

## Abstract

**Background:**

Collision hematologic malignancies, characterized by the concurrent presence of clonally independent myeloid and lymphoid neoplasms within a single patient, are exceptionally uncommon. Diagnosing these malignancies is particularly challenging when they occur in the context of chronic inflammatory diseases, where distinguishing between reactive and neoplastic processes is complex.

**Case presentation:**

We present the case of a 66-years-old male patient with COPD, characterized by a 30 pack-year smoking history, who was admitted due to an acute exacerbation of COPD (AECOPD) accompanied by fever. Notably, despite the complete normalization of C-reactive protein (CRP < 0.2 mg/L), there was a paradoxical increase in the white blood cell (WBC) count to 50.2 × 10^9^/L during treatment with high-dose intravenous methylprednisolone. Concurrently, the absolute lymphocyte count rose to 8.08 × 10^9^/L, a phenomenon we describe as “clinico-laboratory dissociation.” This atypical kinetic pattern prompted further hematologic work-up, including bone marrow evaluation, which revealed the coexistence of chronic-phase chronic myeloid leukemia (CML) [BCR::ABL1 IS: 64.93%; t(9;22)(q34;q11.2)] and chronic lymphocytic leukemia (CLL)-like monoclonal B-cell lymphocytosis (MBL) [CD19^+^CD5^+^CD23^+^CD20(dim), κ-restricted, monoclonal IGH rearrangement]. During the diagnostic process, the patient developed life-threatening *Pneumocystis jirovecii* pneumonia (PJP), confirmed through sputum PCR. The patient received treatment with imatinib (400 mg/day) and high-dose trimethoprim-sulfamethoxazole (TMP-SMX, 15–20 mg/kg/day), resulting in the resolution of respiratory symptoms within 1 week and an early molecular response by day 21, as evidenced by a reduction in BCR::ABL1 IS from 64.93% to 11.03%.

**Conclusion:**

In this patient with COPD undergoing systemic corticosteroid treatment, steroid-resistant lymphocytosis accompanied by WBC–CRP dissociation led to a bone marrow evaluation that revealed two concurrent clonal hematologic disorders. This single case suggests that such a pattern may warrant hematologic assessment in similar clinical settings, and that even subclinical clonal B-cell disorders may carry immunological relevance meriting further study, though this observation requires validation in larger cohorts before informing clinical practice.

## 1 Introduction

Collision hematologic malignancies, characterized by the simultaneous presence of phenotypically and clonally distinct myeloid and lymphoid neoplasms within a single patient, constitute an exceptionally rare diagnostic occurrence. The co-occurrence is likely attributable to genetic predispositions and age-related somatic mutations. Germline variants, such as CHEK2 p.I157T, are known to elevate the risk across both myeloid and lymphoid lineages, whereas somatic clonal hematopoiesis of indeterminate potential (CHIP) can independently drive clonal expansion in either lineage, each with distinct malignancy risk profiles. It remains undetermined whether either mechanism contributed to the concurrent clones observed in this patient, as neither germline sequencing nor CHIP profiling was conducted (1, 2) –a limitation that is addressed in Section “3.6 Limitations.”

In addition to germline and somatic predispositions, chronic inflammatory states may further facilitate clonal expansion. Evidence suggests a bidirectional relationship between chronic obstructive pulmonary disease (COPD) and clonal hematopoiesis. Individuals with clonal hematopoiesis of indeterminate potential (CHIP) exhibit a 1.6-fold increased risk of developing moderate-to-severe COPD and a 2.2-fold increased risk of severe COPD. The prevalence of CHIP in COPD patients is associated with smoking and a history of exacerbations (3). Furthermore, Tet2-deficient mice demonstrate accelerated development of cigarette smoke-induced emphysema (4). The concurrent presence of monoclonal B-cell lymphocytosis (MBL) and CHIP significantly elevates the risk of lymphoid malignancy (hazard ratio [HR] 7.18) compared to either condition alone (MBL: HR 3.48; CHIP: HR 1.89), indicating a potential synergistic interaction (5), which is further explored in Section “3.2 The “common soil” hypothesis: COPD as a shared pro-clonal milieu” concerning the current case. The simultaneous diagnosis of chronic myeloid leukemia (CML) and CLL-like MBL is uncommon, with only a few reported cases, each demonstrating distinct clonal origins (6). The identification of such concurrent malignancies in the context of chronic inflammatory disease is complicated by the challenge of differentiating between neoplastic and reactive leukocytosis, especially in patients undergoing systemic immunosuppressive therapy.

This report details a COPD patient who simultaneously developed CML and CLL-like MBL, identified by a unique “clinico-laboratory dissociation” pattern–marked by increasing lymphocyte-predominant leukocytosis and rising LDH, despite normal CRP levels under high-dose corticosteroids. This pattern, inconsistent with a resolving reactive process, was complicated by life-threatening *Pneumocystis jirovecii* pneumonia (PJP), emphasizing the patient’s heightened immunological risk. The case highlights the importance of recognizing this pattern for prompt hematologic evaluation and the challenges of managing concurrent hematologic malignancies in a COPD-related inflammatory context.

## 2 Case presentation

### 2.1 Patient information

A 66-years-old male with a 30-pack-year smoking history and a previously established diagnosis of chronic obstructive pulmonary disease (COPD) classified as GOLD stage III presented to our institution with a 3-days history of progressively worsening dyspnea, productive cough, and fever, with a peak temperature of 38.6 °C, indicative of an acute exacerbation of COPD (AECOPD). His past medical history was otherwise unremarkable, with no personal or familial history of hematologic malignancies, immunodeficiency disorders, or prior exposure to cytotoxic agents or radiation. Upon physical examination, decreased bilateral breath sounds accompanied by expiratory wheezing were noted. Additionally, there was no evidence of peripheral lymphadenopathy, splenomegaly, or hepatomegaly at the time of admission.

### 2.2 Diagnostic timeline

The principal clinical events and diagnostic milestones are encapsulated in Table 1. In summary, the patient initially received treatment for acute exacerbation of chronic obstructive pulmonary disease (AECOPD) concurrent with an influenza A infection. Despite clinical recovery and normalization of C-reactive protein (CRP) levels, an unanticipated and progressive leukocytosis persists. The morphology and differential counts of peripheral blood were examined, and considering the extent of the abnormality, this prompted bone marrow assessment on the tenth day of hospitalization. This evaluation led to the dual diagnosis of chronic myeloid leukemia (CML) and chronic lymphocytic leukemia-like monoclonal B-cell lymphocytosis (CLL-like MBL). The subsequent onset of *Pneumocystis jirovecii* pneumonia (PJP) in the following week introduced additional diagnostic and therapeutic complexities.

**TABLE 1:** Longitudinal changes in key laboratory parameters during the clinical course.

Variable	Reference range	Jan 9	Jan 16	Jan 26	Feb 5	Feb 22	Feb 27
Hematologic							
White blood cell count (×10^9^/L)	3.5–9.5	13.3	27.5	50.2	27.1	NA	7.3
Neutrophils (%)	40.0–75.0	67.8	84.4	81.7	77.4	NA	65.4
Lymphocytes (%)	20.0–50.0	23.6	12.6	16.1	16.1	NA	25.8
Hemoglobin (g/L)	130–175	147	150	129	139	NA	125
Platelet count (×10^9^/L)	125–350	169	216	216	211	NA	328
Biochemical							
C-reactive protein (mg/L)	0–6.0	2.8	6.2	<0.2	3.5	NA	21.2
Potassium (mmol/L)	3.50–5.30	4.10	3.94	3.09	4.49	NA	4.20
Lactate dehydrogenase (U/L)	0–247	367	414	409	387	NA	212
Molecular							
BCR::ABL1 transcript (IS, %)	0	NA	NA	NA	64.93*	NA	11.03*
*Pneumocystis jirovecii*	Negative	NA	Negative	NA	NA	Positive	NA
*Aspergillus*	Negative	NA	Negative	NA	NA	Negative	NA
*Cryptococcus*	Negative	NA	Negative	NA	NA	Negative	NA

NA, not available; IS, International Scale. *Values on Feb 5 and Feb 27 were taken from bone marrow and peripheral blood.

### 2.3 Detailed clinical course

#### 2.3.1 Initial presentation and infectious phase (hospital days 1–4)

Initial laboratory evaluations indicated a white blood cell (WBC) count of 13.3 × 10^9^/L (reference range: 3.5–9.5 × 10^9^/L), with neutrophils accounting for 67.8% of the total. C-reactive protein (CRP) levels were mildly elevated at 2.8 mg/L. Notably, serum lactate dehydrogenase (LDH) was elevated at 367 U/L (reference range: 0–247 U/L). LDH serves as a non-specific indicator of cellular turnover. At this preliminary stage, before conducting any hematologic analysis or evaluating for *Pneumocystis jirovecii* pneumonia (PJP), the elevation in LDH levels cannot be ascribed to a specific etiology and is therefore documented for ongoing longitudinal assessment. Chest computed tomography (CT) scans revealed emphysematous changes without evidence of new infiltrates or consolidation. Influenza A antigen testing returned positive. The patient was treated with oseltamivir, broad-spectrum antibiotics, and intravenous methylprednisolone at doses up to 80 mg per 24 h. By the fourth day of hospitalization, the patient was afebrile, dyspnea had improved, and inflammatory markers were showing signs of resolution. The initial clinical course was consistent with an infectious acute exacerbation of chronic obstructive pulmonary disease (AECOPD) responding to standard therapeutic interventions.

#### 2.3.2 Emergence of clinico-laboratory dissociation (hospital days 5–10)

**FIGURE 1:**
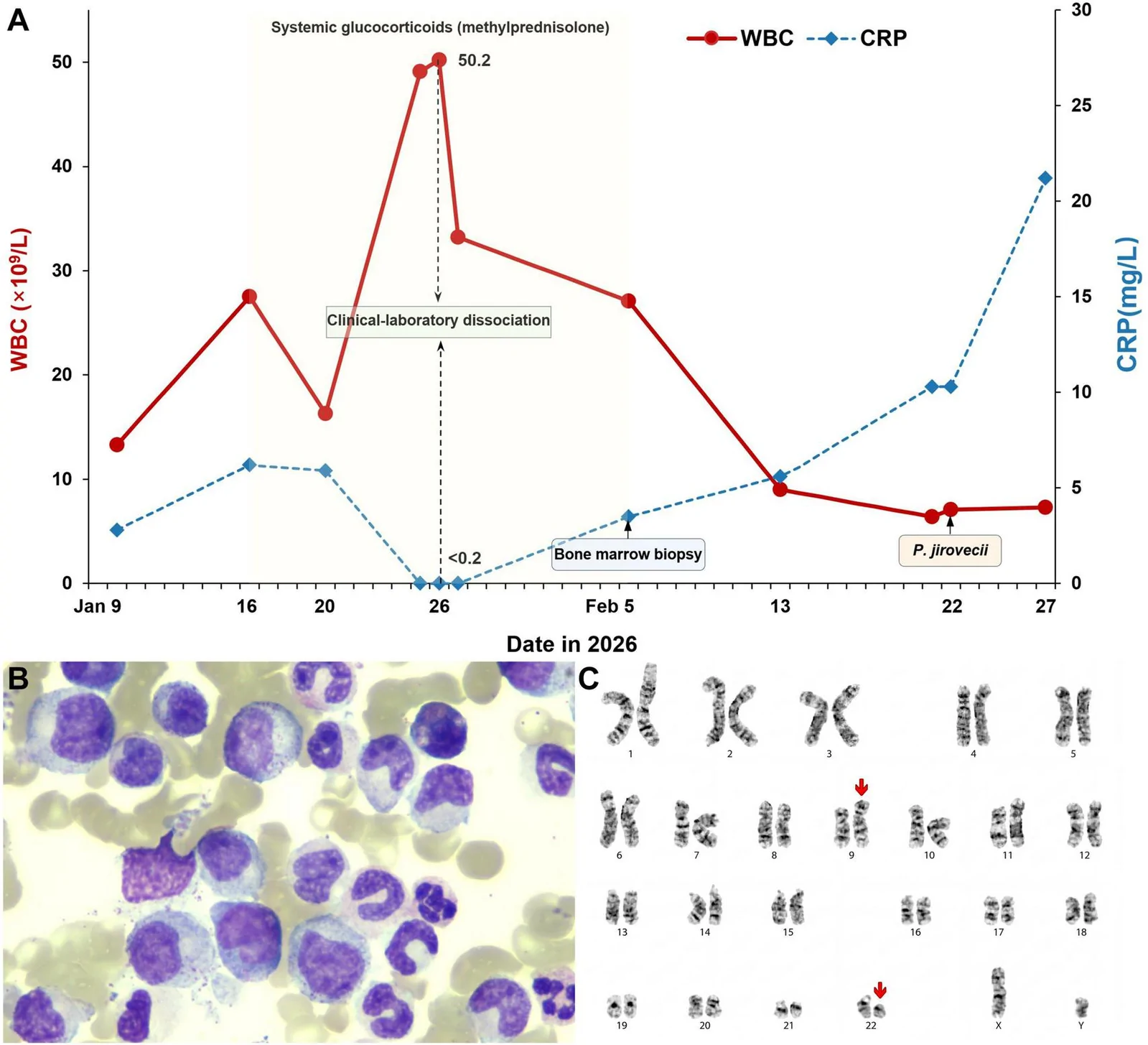
Clinical–laboratory dissociation and hematologic evidence supporting the diagnosis of chronic-phase CML. **(A)** The patient’s clinical timeline shows a “clinico-laboratory dissociation” where, after receiving systemic glucocorticoids for COPD exacerbation, the WBC count rose to 50.2 × 10^9^/L, while CRP levels normalized. It also notes the timing of a bone marrow biopsy and a subsequent *P. jirovecii* infection. **(B)** The bone marrow aspirate smear exhibits significant myeloid hyperplasia, characterized by granulocytic proliferation across various stages of maturation, indicative of chronic-phase chronic myeloid leukemia (×1000). **(C)** The conventional karyotype analysis demonstrates the presence of the Philadelphia chromosome, identified by the reciprocal translocation t(9;22)(q34;q11.2). Red arrows highlight the rearranged chromosomes 9 and 22.

Despite clinical improvement and resolution of fever, serial monitoring of blood counts revealed a progressively and paradoxically increasing leukocytosis (Figure 1A and Table 1). By the tenth day of hospitalization, C-reactive protein (CRP) levels had decreased to less than 0.2 mg/L, indicating a complete resolution of systemic inflammation. However, the white blood cell (WBC) count simultaneously surged to 50.2 × 10^9^/L, with neutrophils constituting 81.7% of the total. Notably, the absolute lymphocyte count increased to 8.08 × 10^9^/L, despite the ongoing administration of high-dose corticosteroid therapy–a potent pharmacological agent typically expected to significantly suppress reactive lymphocyte populations. The phenomenon termed “clinico-laboratory dissociation,” characterized by the full normalization of inflammatory markers concurrent with ongoing lymphocyte proliferation despite high-dose immunosuppression, deviates from the expected dynamics of leukemoid reactions or steroid-induced leukocytosis. These conditions seldom result in white blood cell (WBC) counts exceeding 30 × 10^9^/L and typically resolve following the cessation of the inciting factor. The sustained, steroid-resistant lymphocyte expansion offers persuasive kinetic evidence of an autonomously proliferating clone that is unresponsive to pharmacological treatment, prompting bone marrow assessment. Since peripheral blood flow cytometry to measure the clonal B-cell fraction wasn’t done at this time, we couldn’t directly assess the monoclonal population’s contribution to the total lymphocytosis.

#### 2.3.3 Bone marrow evaluation and dual diagnosis (Feb 5)

**FIGURE 2:**
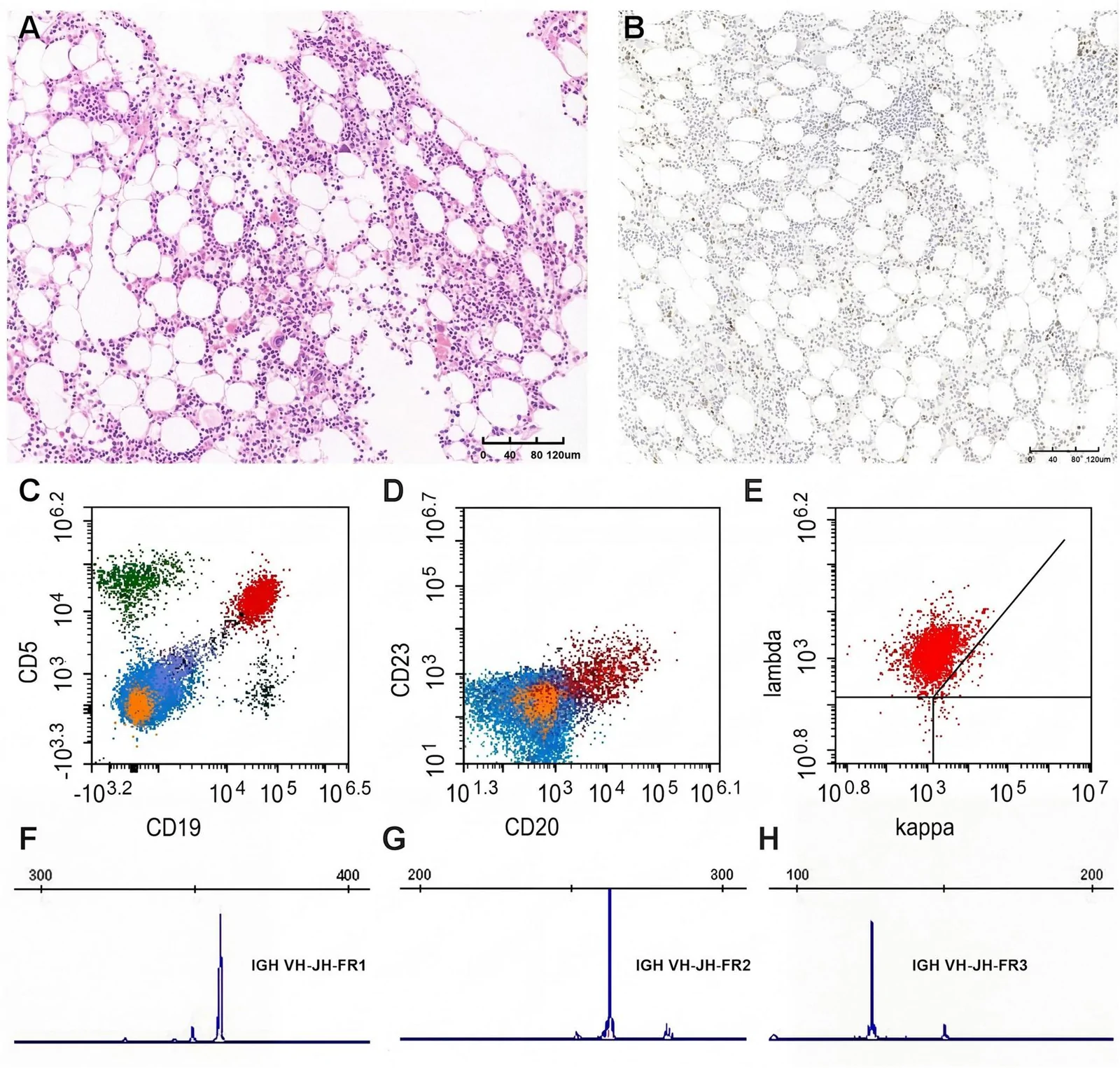
Evidence of simultaneous monoclonal B-cell lymphocytosis in the bone marrow. **(A,B)** The bone marrow biopsy demonstrates focal lymphoid infiltration, which is highlighted by immunohistochemical staining. Scale bars represent 120 μm. **(C–E)** Flow cytometry identifies a minor B-cell population resembling chronic lymphocytic leukemia (CLL), characterized by coexpression of CD19 and CD5, along with expression of CD20 and CD23, and light-chain restriction. **(F–H)** Analysis of IGH gene rearrangement reveals monoclonal peaks in the FR1, FR2, and FR3 regions. Collectively, these findings confirm the presence of coexisting monoclonal B-cell lymphocytosis (MBL).

Bone marrow aspirate smears exhibited pronounced granulocytic hyperplasia with complete maturation and a left-shifted myeloid series, without an increase in blasts (Figure 1B). Conventional cytogenetic analysis using G-banding identified the presence of the Philadelphia chromosome, t(9;22)(q34;q11.2), in all analyzable metaphases (Figure 1C). Quantitative reverse transcription PCR (RT-qPCR) detected the BCR::ABL1 major transcript (e14a2) at a level of 64.93% on the International Scale (IS). These findings are consistent with chronic-phase chronic myeloid leukemia (CML) as classified by the 5th edition of the WHO Classification of Hematolymphoid Tumors (WHO-HAEM5) (7). Concurrently, the bone marrow biopsy revealed normal to mildly increased overall cellularity, predominantly characterized by granulocytic expansion and scattered interstitial small lymphocytic infiltrates (Figures 2A, B). Multiparameter flow cytometry detected an aberrant B-cell population, constituting 2.87% of the total nucleated cells, exhibiting a chronic lymphocytic leukemia (CLL)-like immunophenotype: CD19^+^CD5^+^CD23^+^CD20(dim), with κ light-chain restriction (Figures 2C–E). Capillary electrophoresis fragment analysis (GeneScan) confirmed a clonal immunoglobulin heavy chain (IGH) gene rearrangement in the FR1, FR2, and FR3 regions (Figures 2F–H). Given that the absolute peripheral blood monoclonal B-cell count was significantly below the 5 × 10^9^/L threshold, and in the absence of lymphadenopathy or organomegaly, these findings met the diagnostic criteria for CLL-like monoclonal B-cell lymphocytosis (MBL) according to both the WHO-HAEM5 and the International Consensus Classification (ICC) (8, 9).

#### 2.3.4 Development of PJP and combined treatment (Feb 5–27)

During the diagnostic assessment, the patient exhibited a significant febrile response, with a temperature reaching 39.2 °C, alongside acute hypoxemic respiratory failure, indicated by an SpO_2_ level of 82% while breathing ambient air. The sputum fungal nucleic acid triplex assay, targeting *Pneumocystis*, *Aspergillus*, and *Cryptococcus neoformans*, yielded a positive result for *Pneumocystis jirovecii*. Consequently, a definitive diagnosis of *Pneumocystis jirovecii* pneumonia (PJP) was established in accordance with the guidelines outlined in the “Expert Consensus on the Prevention and Treatment of PJP in Patients with Hematological Malignancies in China” (10). A comprehensive treatment regimen was initiated simultaneously, consisting of imatinib at a dosage of 400 mg daily for chronic myeloid leukemia (CML), high-dose trimethoprim-sulfamethoxazole (TMP-SMX) administered intravenously at 15–20 mg/kg/day for *Pneumocystis jirovecii* pneumonia (PJP) in accordance with ECIL-6 guidelines (11), and active surveillance for monoclonal B-cell lymphocytosis (MBL). The patient exhibited rapid clinical improvement, evidenced by the resolution of respiratory failure and normalization of white blood cell count within 1 week. By Feb 27, BCR::ABL1 transcript levels had decreased from 64.93% to 11.03% on the International Scale (IS), indicating a favorable trend toward early molecular response (EMR). According to the 2025 update on CML management, formal EMR assessment is standardized at 3 months (12). Previous landmark studies have demonstrated that a BCR::ABL1 IS greater than 10% at 3 months is associated with significantly poorer 8-years overall survival (56.9% compared to 93.3%; *P* < 0.001) (13) and 5-years overall survival (87% compared to 97% for IS ≤ 1%) (14), highlighting the critical importance of close molecular monitoring in this patient.

### 2.4 Key ancillary findings

Upon admission, a nasopharyngeal swab tested positive for influenza A antigen, and lactate dehydrogenase (LDH) levels were mildly elevated at presentation (367 U/L, Day 1). On January 16, a sputum fungal nucleic acid triplex assay was performed, specifically targeting *Pneumocystis jirovecii*, *Aspergillus*, and *Cryptococcus neoformans*, at which point LDH levels had increased to 414 U/L. The assay results were negative for all three pathogens, including *P. jirovecii*, as detailed in Table 1. Subsequently, LDH levels gradually declined (409 U/L on Day 18; 387 U/L on Day 28) and normalized to 212 U/L by Day 50, coinciding with the clinical resolution of both the hematologic malignancy and the opportunistic infection. These findings suggest the absence of clinically overt *Pneumocystis jirovecii* pneumonia (PJP) at the time of the Day 7 assay. However, given that the test was conducted 6 days post-admission and sputum-based nucleic acid testing is less sensitive than bronchoalveolar lavage, the possibility of subclinical *P. jirovecii* colonization or early infection present at admission cannot be entirely excluded. No additional fungal testing was conducted between January 16 and the eventual positive result on February 22.

### 2.5 Laboratory trends

Table 1 presents the longitudinal alterations in essential laboratory parameters. Figure 1 illustrates the trajectory of white blood cell (WBC) count and C-reactive protein (CRP) levels throughout the clinical course, with a focus on the period between hospital days 5 and 10. During this interval, the divergence between decreasing CRP levels and increasing leukocytosis is most pronounced, exemplifying the phenomenon known as “clinico-laboratory dissociation.”

## 3 Discussion

### 3.1 The “clinico-laboratory dissociation” as a diagnostic clue in this case

In this case, a key diagnostic clue was an unusual kinetic pattern of WBC and lymphocyte counts (Table 1) that didn’t align with reactive hematologic processes: increasing leukocytosis and steroid-resistant lymphocyte expansion, despite normal CRP levels. LDH was slightly elevated at 367 U/L initially and increased to 414 U/L by day 7, before diagnosing hematologic malignancy or PJP. A sputum PCR for *Pneumocystis jirovecii* was negative, but a low-level infection couldn’t be ruled out due to sputum testing’s lower sensitivity compared to bronchoalveolar lavage. Therefore, the LDH increase likely indicates a mix of myeloproliferative activity and possible subclinical infection, rather than just clonal proliferation.

The key evidence was the steroid-resistant lymphocytosis itself. Normally, high-dose methylprednisolone causes lymphopenia, not an increase in lymphocytes. This unexpected rise suggested an autonomously proliferating lymphoid clone, unlike a leukemoid reaction or steroid-induced neutrophilia. We recognize that this interpretation relies on total lymphocyte count kinetics rather than direct measurement of the clonal B-cell fraction in blood. The specific contribution of the CLL-like MBL clone to the 8.08 × 10^9^/L peak lymphocytosis wasn’t quantified, and a reactive T-cell or NK-cell component might also be present. We propose that in COPD patients under systemic immunosuppression, a dissociation between WBC and CRP levels, along with steroid-resistant lymphocytosis, warrants further hematologic investigation, even if the acute illness seems resolved. This pattern isn’t a new diagnostic finding and doesn’t inherently require a bone marrow exam. Typically, the initial approach involves peripheral blood analysis, differential counts, flow cytometry, and molecular testing. However, in this patient, the severity and persistence of the abnormality justified moving directly to a marrow evaluation. This observation, based on a single case, needs further validation.

### 3.2 The “common soil” hypothesis: COPD as a shared pro-clonal milieu

This case explores the “common soil” hypothesis, suggesting that distinct clonal malignancies can develop in the same inflammatory environment without a shared genetic origin. Here, a BCR::ABL1-driven myeloid clone and a CLL-like B-cell clone, are presumed to represent clonally independent processes, based on their distinct immunophenotypic and cytogenetic features, similar to a previous case of concurrent CML and MBL (6). Their apparent simultaneous occurrence in a patient with COPD-related inflammation implies a shared permissive environment, although Direct molecular confirmation of clonal independence, such as the BCR::ABL1 status of the MBL clone, was not conducted in this case. The link between clonal hematopoiesis and COPD, along with the combined risk of MBL and CHIP, supports the idea that COPD-associated inflammation could facilitate such malignancies without a common genetic basis (3–5).

A significant limitation of this study is the absence of formal germline variant profiling, such as for CHEK2 p.I157T, and comprehensive clonal hematopoiesis of indeterminate potential (CHIP) mutation analysis in this patient. This limitation is the primary reason the discussion is presented as hypothesis-generating rather than confirmatory. Consequently, the “common soil” hypothesis in this context is currently supported solely by indirect clinical and epidemiological evidence, lacking direct molecular validation. Future research on collision hematologic malignancies should incorporate comprehensive germline sequencing and high-sensitivity CHIP profiling to rigorously assess this hypothesis.

### 3.3 The “triple-hit” immunological vulnerability and PJP risk

The severe *Pneumocystis jirovecii* pneumonia (PJP) in this patient highlights the “triple-hit” immunological vulnerability caused by concurrent clonal hematopoietic disorders and chronic inflammatory conditions requiring systemic immunosuppression. Three interacting factors were identified: (1) COPD-related structural and innate immune impairment, which weakens lung defenses and alveolar immunity, increasing infection risk; (2) immune dysregulation from clonal B-cell disease–bone marrow flow cytometry at diagnosis showed a skewed lymphocyte distribution favoring B cells over T cells (B cells 61.31%, T cells 27.43%), a pattern consistent with, but not diagnostic of, functional immunodeficiency as reported in CLL (15); notably, this assessment was limited to bone marrow and was not corroborated by peripheral immunoglobulin or CD4^+^/CD8^+^ measurements (see Section “3.6 Limitations”); (3) iatrogenic immunosuppression from high-dose systemic corticosteroids for AECOPD, a known PJP risk factor in hematologic malignancies (11).

The convergence of these three factors may have created greater immunological vulnerability than any single factor alone, however, the isolated impact of low-count MBL on PJP development cannot be determined from a single case due to the influence of COPD-related immune issues and corticosteroid use. This observation generates a hypothesis, suggesting future research should explore PJP risk models that include subclinical clonal hematopoietic disorders like MBL, instead of just focusing on corticosteroid dosage and duration. Current ECIL-5 TMP-SMX guidelines, based on corticosteroid dose and duration (>20 mg/day for over 4 weeks), reduce PJP incidence by 91% but don’t consider underlying clonal B-cell disorders (16). Applying these guidelines to subclinical MBL and altering prophylaxis timing with corticosteroid initiation needs specific prospective validation and can’t be concluded from just this case.

### 3.4 Implications for CML management in the context of concurrent MBL

Imatinib, at 400 mg daily, was selected as the initial TKI therapy for CML following current guidelines (12). A decrease in BCR::ABL1 IS from 64.93% to 11.03% by day 21–a 4.8-fold reduction–suggests a promising early response; however, a formal EMR evaluation at 3 months is essential. The presence of CLL-like MBL does not affect TKI choice or CML monitoring, though the impact of imatinib on the B-cell clone was not evaluated and remains to be studied. As per WHO-HAEM5 and ICC guidelines, MBL is being actively monitored with serial peripheral blood immunophenotyping and lymph node examination (8, 9).

### 3.5 Differential diagnosis

Table 2 presents a structured differential diagnostic analysis. The primary diagnostic considerations encompassed leukemoid reaction, steroid-induced leukocytosis, chronic myeloid leukemia (CML) with reactive lymphocytosis, and chronic lymphocytic leukemia/small lymphocytic lymphoma (CLL/SLL) with secondary neutrophilia. As detailed above (Section “3.1 The “clinico-laboratory dissociation” as a diagnostic clue in this case”), leukemoid reaction and steroid-induced leukocytosis were excluded based on the clinico-laboratory dissociation pattern. CLL/SLL was differentiated from monoclonal B-cell lymphocytosis (MBL) by maintaining an absolute peripheral blood B-cell count below 5 × 10^9^/L, with no evidence of lymphadenopathy or organomegaly, in strict adherence to the WHO-HAEM5 and International Consensus Classification (ICC) diagnostic criteria (8, 9). The diagnosis of CML was unequivocally confirmed by the detection of the t(9;22)(q34;q11.2) translocation in 100% of metaphases and a BCR::ABL1 International Scale (IS) value of 64.93%.

**TABLE 2:** Diagnostic timeline and key decision points.

Date	Clinical event	Key laboratory/imaging findings	Diagnostic consideration	Action taken
9-Jan	Admission: AECOPD and fever	WBC 13.3 × 10^9^/L; CRP 2.8 mg/L; LDH 367 U/L; influenza A (+); CT: emphysema, no infiltrates	AECOPD + influenza A	Oseltamivir, antibiotics, methylprednisolone ≤ 80 mg/24 h
13-Jan	Fever resolved; respiratory improvement	Not available	AECOPD resolving	Continue treatment
16-Jan	Unexpected leukocytosis	WBC 27.5 × 10^9^/L; CRP 6.2 mg/L; LDH 414 U/L	Leukemoid reaction vs. steroid effect	Watchful monitoring
26-Jan	Clinico-laboratory dissociation identified	WBC 50.2 × 10^9^/L; CRP < 0.2 mg/L; ALC 8.08 × 10^9^/L; LDH 409 U/L	Autonomous clonal proliferation suspected	Urgent bone marrow biopsy
5-Feb	Bone marrow results; acute respiratory deterioration	BCR::ABL1 64.93% IS; t(9;22) in all metaphases; CLL-like MBL by flow cytometry/IGH;	CML + CLL-like MBL	Imatinib 400 mg/day
22-Feb	Persistent fever	*Pneumocystis jirovecii*: positive (cycle threshold, CT: 34.08)	*Pneumocystis jirovecii* pneumonia (PJP)	TMP-SMX 15–20 mg/kg/day
25-Feb	Respiratory resolution; WBC normalizing	WBC 9.2 × 10^9^/L; SpO_2_ > 96% on room air	Treatment response	Continue imatinib; TMP-SMX completing course
27-Feb	Early molecular response	BCR::ABL1 11.03% IS	Favorable early molecular response trend	Continue imatinib; MBL active surveillance

### 3.6 Limitations

This case study has several limitations: (1) as noted in Section “3.2 The “common soil” hypothesis: COPD as a shared pro-clonal milieu,” the lack of germline and CHIP profiling prevents assessing a shared molecular basis for the malignancies; (2) the BCR::ABL1 status of the MBL B-cell clone was not directly analyzed, leaving its clonal origin unconfirmed; (3) the short follow-up period limits understanding of long-term interactions among CML under TKI therapy, MBL progression risk, and COPD management. Future systematic CHIP and germline profiling in similar cases is needed to clarify the molecular structure of these hematologic malignancies. (4) As discussed in Section “3.3 The “triple-hit” immunological vulnerability and PJP risk,” the immune assessment was restricted to bone marrow flow cytometry at diagnosis, without peripheral serum immunoglobulin or CD4^+^/CD8^+^ measurements, leaving the MBL-related immune dysregulation aspect of the “triple-hit” hypothesis unverified at the peripheral level. (5) As noted in Section “3.1 The “clinico-laboratory dissociation” as a diagnostic clue in this case,” *P. jirovecii* testing was only conducted on day 7 before the confirmed PJP diagnosis on day 45, so a minor infection contributing to early LDH elevation cannot be completely ruled out. The peak lymphocytosis peripheral blood monoclonal B-cell count wasn’t measured by flow cytometry, so the idea that autonomous clonal expansion is the main cause of the lymphocytosis is inferred, not proven.

## 4 Conclusion

This case illustrates those hematologic malignancies, specifically the concurrent occurrence of chronic myeloid leukemia (CML) and chronic lymphocytic leukemia-like monoclonal B-cell lymphocytosis (CLL-like MBL), can develop within a pro-inflammatory environment driven by chronic obstructive pulmonary disease (COPD). These malignancies may remain clinically undetected until revealed by a distinctive pattern of “clinico-laboratory dissociation.” This report presents three insights that need validation in larger studies before influencing clinical practice: (1) In COPD patients on corticosteroids, if WBC-CRP dissociation and steroid-resistant lymphocyte expansion are suspected, start with hematological tests and consider a bone marrow examination if needed; (2) the presence of myeloid and lymphoid clones, presumed to be clonally independent (though not molecularly confirmed), suggests a “common soil” model in which COPD-related inflammation allows multi-lineage clonal expansion, warranting future CHIP/germline profiling; (3) the combination of COPD-related immune impairment, clonal B-cell dysregulation, and corticosteroid-induced immunosuppression may increase the risk of opportunistic infections like PJP, a hypothesis that requires prospective confirmation before guiding prophylaxis decisions.

## 5 Patient perspective

The patient characterized the clinical trajectory as both alarming and perplexing, particularly due to the unexpected deterioration in blood test results occurring concurrently with an improvement in respiratory symptoms. He experienced significant distress over the rapid escalation of his leukocyte count and the subsequent onset of severe respiratory difficulties necessitating intensive respiratory support. The concurrent diagnosis of two hematological malignancies was psychologically overwhelming, necessitating time for him to comprehend the implications of each condition and their respective management strategies. Upon the initiation of targeted therapy with imatinib and the successful treatment of the pulmonary infection, the patient reported substantial physical improvement and expressed relief at having a coherent explanation and a definitive treatment plan. He indicated that the clarity of the diagnosis–despite its inherent complexity–provided reassurance, and he demonstrated a strong commitment to adhering to long-term imatinib therapy and the recommended monitoring program for monoclonal B-cell lymphocytosis (MBL).

## Data Availability

The raw data supporting the conclusions of this article will be made available by the authors, without undue reservation.
